# Interaction Analysis Reveals Complex Genetic Associations with Alzheimer’s Disease in the *CLU* and *ABCA7* Gene Regions

**DOI:** 10.3390/genes14091666

**Published:** 2023-08-23

**Authors:** Alireza Nazarian, Brandon Cook, Marissa Morado, Alexander M. Kulminski

**Affiliations:** Biodemography of Aging Research Unit, Social Science Research Institute, Duke University, Durham, NC 27705, USAmarissa.morado@duke.edu (M.M.)

**Keywords:** dementia, Aging, SNPs interaction, compound genotype, genetic heterogeneity

## Abstract

Sporadic Alzheimer’s disease (AD) is a polygenic neurodegenerative disorder. Single-nucleotide polymorphisms (SNPs) in multiple genes (e.g., *CLU* and *ABCA7*) have been associated with AD. However, none of them were characterized as causal variants that indicate the complex genetic architecture of AD, which is likely affected by individual variants and their interactions. We performed a meta-analysis of four independent cohorts to examine associations of 32 *CLU* and 50 *ABCA7* polymorphisms as well as their 496 and 1225 pair-wise interactions with AD. The single SNP analyses revealed that six *CLU* and five *ABCA7* SNPs were associated with AD. Ten of them were previously not reported. The interaction analyses identified AD-associated compound genotypes for 25 *CLU* and 24 *ABCA7* SNP pairs, whose comprising SNPs were not associated with AD individually. Three and one additional *CLU* and *ABCA7* pairs composed of the AD-associated SNPs showed partial interactions as the minor allele effect of one SNP in each pair was intensified in the absence of the minor allele of the other SNP. The interactions identified here may modulate associations of the *CLU* and *ABCA7* variants with AD. Our analyses highlight the importance of the roles of combinations of genetic variants in AD risk assessment.

## 1. Introduction

Sporadic late-onset Alzheimer’s disease (AD), the most common cause of dementia in the United States and worldwide, is a multifactorial polygenic disorder. Age and genetic factors are the two major determinants of AD risk, and modifiable cardiovascular and lifestyle factors are also deemed to have some roles in AD development [1]. The apolipoprotein E (*APOE*) ε2 and ε4 alleles, with protective and adverse effects, respectively, are the main AD-associated genetic factors [2,3,4]. Additionally, multiple variants and genes outside of the *APOE* 19q13.3 locus have been associated with AD in recent years [5,6].

*CLU* (Clusterin) and *ABCA7* (Adenosine triphosphate Binding Cassette subfamily A member 7) genes located on chromosomes 8p21.1 and 19p13.3, respectively, are two of these genes, which are subjects of our study [5,7,8,9]. The *CLU* gene encodes a protein, which plays roles in cell survival and death, inflammatory responses, and lipid transport [7,8]. Genome-wide association studies (GWAS) have thus far identified AD associations of several single-nucleotide polymorphisms (SNPs) mapped to this gene, such as rs11787077 [10], rs867230 [11], and rs9331896 [12,13,14]. While elevated levels of CLU protein were detected in the brain and cerebrospinal fluid (CSF) of AD-affected subjects, functional studies have revealed the dual function of *CLU* as it may have both neuroprotective and neurodegenerative effects [8]. For instance, depending on determinant factors like the ratio of extracellular to intracellular CLU protein, the ratio of CLU to Aβ, hypoxia-induced stress, etc., the *CLU* gene may facilitate or reduce amyloid β (Aβ) clearance, affecting Aβ aggregation [8,15,16]. 

*ABCA7* gene encodes a transmembrane transporter, which is involved in lipid homeostasis [7,9]. The top three AD-associated SNPs of this gene reported by previous GWAS are rs12151021 [10,11,17], rs3764650 [18], and rs4147929 [12,13,14]. *ABCA7* variants have also been linked to increased Aβ deposition and amyloid plaque formation [19,20], gray matter density, and hippocampus asymmetry in the brains of cognitively impaired subjects [21,22]. AD mouse model experiments have shown that *Abca7* knockout may increase Aβ production and decrease Aβ clearance due to impairment of phagocytosis of Aβ aggregates by microglia and macrophages [23,24]. 

None of the AD-associated SNPs identified thus far were described as causal factors. Instead, all studies report some degree of incomplete penetrance that is consistent with the complex genetic architecture of AD (e.g., haplotypes and interactions). There is a multitude of evidence of such a complex genetic landscape within the *APOE* 19q13.3 locus [25,26,27,28,29,30,31,31,32,33,34,35,36,37]. For instance, we have shown that the linkage disequilibrium (LD) patterns in this locus are significantly different in the AD-affected subjects and AD-unaffected controls [30,31,32,37]. Our previous studies also reported efficient modulation of the associations of the *APOE* ε2 and ε4 alleles with AD by variants from the *TOMM40* and *APOC1* genes [33,34,37].

Here, we hypothesized that similar to the *APOE* locus, SNPs within either *CLU* or *ABCA7* gene cluster can jointly impact the risk of AD. We examined associations of 32 *CLU* SNPs and 50 *ABCA7* SNPs with AD and their pair-wise interactions in each cluster separately. Interaction analysis leveraged models with compound genotypes represented by combinations of genotypes from SNP pairs and models with multiplicative SNP-by-SNP interaction terms using data from four independent AD studies. Our analyses identified novel AD-associated interactions for 25 SNP pairs in the *CLU* locus and 24 SNP pairs in the *ABCA7* locus, whose comprising SNPs were not significantly associated with AD individually. In addition, we showed that significant associations of individual SNPs with AD could be significantly modulated by the other SNP in three *CLU* SNP pairs and one *ABCA7* SNP pair in such a way that the effect of the minor allele of one SNP in each pair became stronger in the absence of the minor allele of the other SNP.

## 2. Methods

### 2.1. Study Participants

We analyzed genotype and phenotype data on subjects from the following four datasets: three Alzheimer’s Disease Centers (ADCs) data from the Alzheimer’s Disease Genetics Consortium (ADGC) initiative [38], whole-genome sequencing data from the Alzheimer’s Disease Sequencing Project (ADSP-WGS) [39,40], National Institute on Aging (NIA)’s Late-Onset Alzheimer’s Disease Family-Based Study (LOAD-FBS) [41,42], and the United Kingdom Biobank data (UKB) [43]. To enhance statistical power, our analyses focused on subjects of Caucasian ancestry, as they constituted the vast majority of the samples. Those ADSP-WGS subjects who were in common with ADGC and LOAD-FBS were excluded to keep datasets independent. In addition, the unaffected UKB subjects younger than 65 years were excluded to age-match the case and control UKB sets. In the cohorts under consideration, the AD affection status was mainly determined through clinical assessment adhering to the National Institute of Neurological and Communicative Disorders and Stroke and the AD and Related Disorders Association (NINCDS-ADRDA) guidelines. This process involved the use of various tools, including, for example, the Cognitive Assessment Battery, which evaluated cognitive function through measures such as story recall score, digit span forward/backward score, word-finding score, etc. [42,44,45]. The AD status of subjects was directly reported by the ADGC, ADSP-WGS, and LOAD-FBS primary investigators. AD cases in UKB were reported in the form of ICD-10 (International Classification of Disease Codes, 10th revision) codes. Table S1 contains basic information about the study participants.

### 2.2. Genotype Data and Quality Control (QC)

Two sets of 1786 and 378 SNPs in *ABCA7* and *CLU* genes were selected, respectively, from those available in International Genomics of Alzheimer’s Project (IGAP) stage 1 analyses in Lambert et al. study [12]. For the *CLU* gene, we selected SNPs within approximately 60 kb up-/downstream of the lead SNP rs4236673. For the *ABCA7* gene, we selected SNPs within approximately 550 kb downstream and 220 kb upstream of the lead SNP rs4147929. Larger distances were used here to include *BSG* (downstream of *ABCA7*) and *STK11* (upstream of *ABCA7*) genes, which have been reported among AD-associated loci in the GWAS catalog [6]. 

SNPs with minor allele frequencies below 5%, Hardy–Weinberg *p*-values less than 1 × 10^−6^, missing rates above 5%, or imputation quality lower than r^2^ = 0.9, as well as subjects with missing rates above 5%, were filtered out. In addition, SNPs in each set were pruned considering their LD measures so that no SNP pair had LD greater than r^2^ = 0.7. This was performed using PLINK (v2.0) (www.cog-genomics.org/plink/2.0/ (accessed on 25 June 2023)) [46]. These resulted in two subsets of 32 and 50 SNPs for *CLU* and *ABCA7* genes, respectively, which were subject to our genetic analyses. Basic information on SNPs used in our genetic analyses is provided in Table S2.

### 2.3. Analysis of the AD Risk

We performed three types of analyses of the associations between AD (categorized as presence or absence of the AD diagnosis) and genetic variants in *CLU* and *ABCA7* genes separately in each of the selected studies, as detailed below. A dominant allelic-effect at each locus (i.e., heterozygote and minor allele homozygote genotypes having the same effects) was consistently used in all analyses to offset an issue of small samples of minor allele homozygotes for some SNPs. For all analyses, we used stats (v4.3.0) [47] and lme4 (v1.1.34) [48] packages in R (v4.3.0) [47] adjusting the models for age and sex of subjects, rs7412 and rs429358 genotypes (i.e., *APOE* ε2 and ε4 encoding SNPs) as fixed-effects covariates as well as family-ID as a random-effects covariate in the case of LOAD-FBS dataset, which has considerable family structure.

#### 2.3.1. The Analysis of Compound Genotypes (CompG)

The analysis of compound genotypes (CompG) was focused on identifying the associations between AD and CompG constructed from SNP pairs at each of *CLU* (496 SNP pairs) and *ABCA7* (1225 SNP pairs) genes separately. For any SNP pair, a CompG with four distinct factor levels was obtained. The coding schema for the dominant genetic model used for construing CompG is shown in Table 1.

In our models, the MM compound genotype (i.e., major allele homozygotes at both SNP_1_ and SNP_2_) was the reference factor level to which the significance of the effects of the other three levels was compared. We further examined the differences between the effect sizes for any pair of Mm, mM, and mm compound genotypes (i.e., mM-Mm, mM-mm, and Mm-mm differences) using a chi-square test with one degree of freedom [49]:

$$\chi^{2}=\frac{{\left(b_{1}-{\;b}_{2}\right)}^{2}}{{se}_{1}^{2}+{se}_{2}^{2}}\;$$

Here, *b*_1_ (*se*_1_) and *b*_2_ (*se*_2_) are the β coefficients (their standard errors) obtained from meta-analyses for the two CompG levels of interest.

#### 2.3.2. Single SNP Analysis

Single SNP analysis was performed to examine the associations between AD risk and each of the 32 *CLU* and 50 *ABCA7* SNPs. We compared SNP effects from single SNP models with CompG effects using the aforementioned chi-square test when significant CompG comprised of one or two significantly AD-associated SNP(s). 

#### 2.3.3. Traditional Interaction Analysis

Traditional interaction analysis for each SNP pair under consideration was performed through fitting interaction models where both SNPs and their interaction term were included. 

The association results from four analyzed cohorts were combined using a fixed-effects inverse-variance meta-analysis using the metafor (v4.2.0) [50] package in R (v4.3.0) [47]. Significant findings from our meta-analyses or the chi-square tests of the differences in the effects were determined at a false-discovery rate (FDR) adjusted P_FDR_ < 0.05 [51,52]. Volcano plots were depicted using ggplot2 (v3.4.2) [53], tidyverse (v2.0.0) [54], and ggrepel (v0.9.3) [55] packages in R (v4.3.0) [47].

## 3. Results

Detailed results from our meta-analyses of the associations between AD and CompG in *CLU* and *ABCA7* genes, as well as the results from interaction models, are summarized in Tables S3–S6. Additionally, color-coded LD matrices for the SNPs selected within the *CLU* and *ABCA7* gene regions are provided in Tables S7 and S8, respectively. Detailed LD information about significant SNP pairs has been shown in Tables S9 and S10.

### 3.1. CLU Gene Results

**Figure 1:**
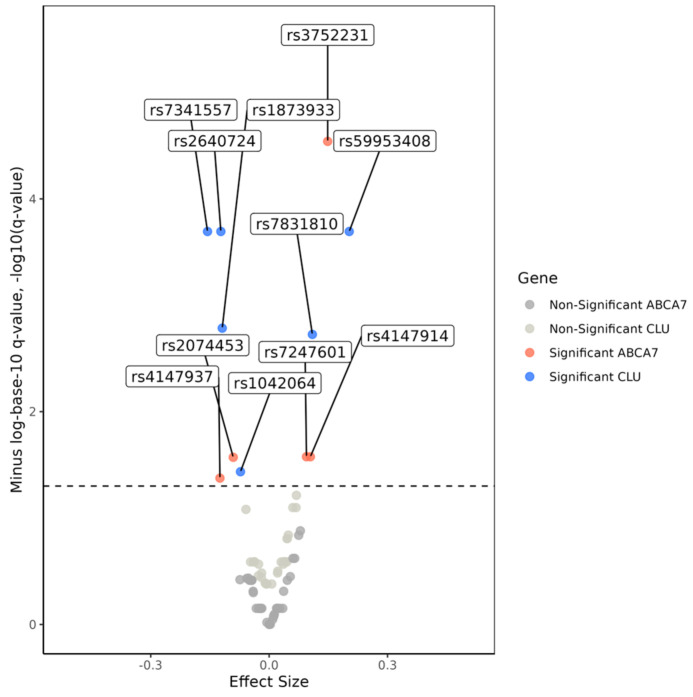
Volcano plot for the associations between Alzheimer’s disease (AD) and the 32 *CLU* and 50 *ABCA7* single-nucleotide polymorphisms (SNPs). The x-axis displays effect sizes for SNPs, while the y-axis shows minus-logarithm-base-10-transformed *q*-values, representing false-discovery rate (FDR) adjusted significance. The dashed line indicates the significance threshold, set at −log10 (*q*-value = 0.05) = 1.3. Above this cutoff line, blue and red dots represent AD-associated SNPs in the *CLU* and *ABCA7* genes, respectively. Non-significant SNPs in these two genes are depicted in light and dark gray, respectively. Numerical estimates are provided in Table 2, Tables S3–S6.

We found that six of 32 *CLU* SNPs were associated with AD risk in the meta-analyses of the results from single SNP models at P_FDR_ < 0.05 (Table 2, Tables S3 and S4 and Figure 1). Minor alleles of four of these SNPs were negatively associated, and those of the other two were positively associated with the AD risk. Their pair-wise LD measured by |r| was relatively small, ranging from 9.12 × 10^−5^ to 0.660 in the AD-affected group and from 0.007 to 0.649 in the AD-unaffected group (Table S9). 

In addition, our meta-analyses identified AD-CompG associations of 169 of 496 SNP pairs corresponding to the *CLU* gene at P_FDR_ < 0.05. Three additional SNP pairs (i.e., rs66924402−rs59953408, rs1042064−rs7831810, rs7341557−rs7831810) had significant ‘mM-Mm’ differences while their CompGs were not associated with AD. Four of five SNPs that defined these three SNP pairs were associated with AD in the single SNP models. None of these 172 SNP pairs had significant interaction terms in the meta-analysis of the results from traditional interaction models (Tables S3 and S4).

**Figure 2:**
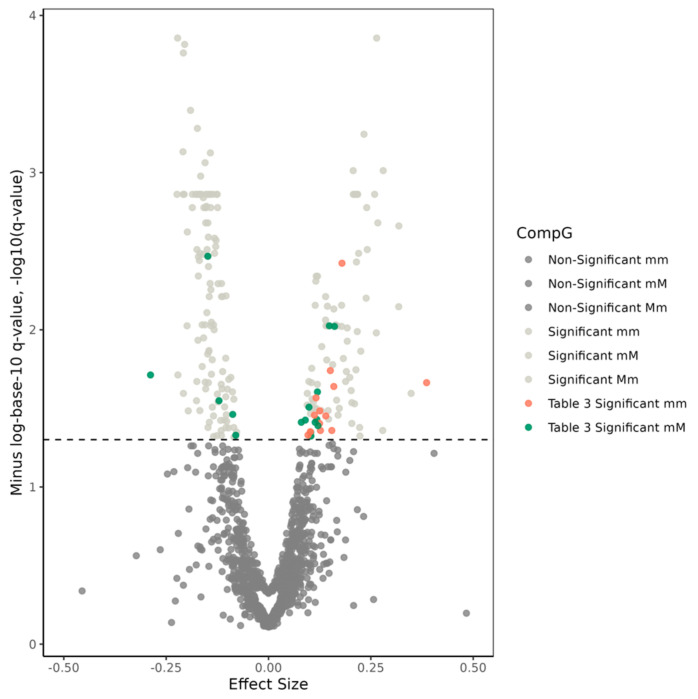
Volcano plot for compound genotype (CompG) analyses of the 496 single-nucleotide polymorphism (SNP) pairs selected within the *CLU* gene region. The x-axis displays effect sizes of CompGs, while the y-axis shows minus-logarithm-base-10-transformed *q*-values, representing false-discovery rate (FDR) adjusted significance. The dashed line indicates the significance threshold, set at −log10 (*q*-value = 0.05) = 1.3. Dark-gray dots below the cutoff line represent non-significant effects for Mm, mM, and mm CompGs. Light-gray dots above the cutoff line indicate significant SNP pairs whose comprising SNPs were associated with Alzheimer’s disease (AD) individually. Red and green dots above the cutoff line denote SNP pairs whose comprising SNPs were not associated with AD individually and were presented in Table 3 and Table S3.

Twenty-five of 172 significant SNP pairs were composed of 18 SNPs that were not associated with AD in the single SNP meta-analyses (P_FDR_ ≥ 0.05). Of these 25 pairs, 12 pairs had significant mM effects, nine pairs had significant mm effects, and four pairs had significant mM and mm effects (Table 3 and Table S3 and Figure 2). One of these SNP pairs (i.e., rs17466684-rs9331888) had a significant ‘mM-Mm’ difference as assessed using a chi-square test (*p* = 1.09 × 10^−3^, P_FDR_ = 2.46 × 10^−2^). This difference was not attributable to the differences in the main effects of rs17466684 and rs9331888 in the single SNP models (*p* = 1.06 × 10^−1^ from comparing their main effects using the chi-square test). Instead, it was elucidated by an interaction, where the effect of the minor allele of rs17466684 became significant in the absence of carriers of the minor allele of rs9331888 (i.e., mM genotype). 

LD measured by |r| for these 25 SNP pairs ranged between 0.008 and 0.704 in the AD-affected group and between 0.013 and 0.667 in the AD-unaffected group. Additionally, six of these 25 pairs had significantly different LD between the AD-affected and unaffected groups at Bonferroni-adjusted *p* < 0.002 (i.e., 0.05/25) in the chi-square test (five with larger and one with smaller LD magnitudes in the AD-affected group) (Table S10). 

One or both SNPs that defined each of the remaining 147 significant SNP pairs were associated with AD (P_FDR_ < 0.05) in the single SNP models (Table S4 and Figure 2). To determine if the significant CompG effects in these 147 SNP pairs were statistically different from the effects of their comprising AD-associated SNP(s), we compared the CompG β coefficients with the corresponding SNP main effects from the single SNP models using the chi-square test. These tests showed that the significance of CompG effects can be justified based on the significance of the main effects of SNPs (P_FDR_ ≥ 0.05 in the comparison tests). Additionally, 33 of these 147 pairs had significant ‘mM-Mm’ differences (P_FDR_ < 0.05 in the chi-square test) (Table S4). For 30 of 33 SNP pairs, these differences were explained by the main effects of the comprising SNPs in the single SNP models. For three pairs—rs114072046−rs59953408, rs2640724−rs881146, and rs59953408−rs17057419—, however, the ‘mM-Mm’ differences were affected by SNP interactions. This is evidenced by smaller *p*-values for the ‘mM-Mm’ differences (*p* = 6.78 × 10^−4^, 8.68 × 10^−4^, and 2.87 × 10^−3^, respectively) than for the differences of the main effects of the comprising SNPs (*p* = 1.13 × 10^−3^, 2.09 × 10^−3^, and 5.05 × 10^−3^, respectively from comparing the main effects of the corresponding SNPs in the single SNP models using the chi-square test). The LD r coefficients for these three pairs were 0.190, 0.277, and 0.167, respectively, in the AD-affected group and 0.174, 0.241, and 0.122, respectively, in the AD-unaffected group (Table S7).

### 3.2. ABCA7 Gene Results

Our meta-analyses revealed that 5 of 50 *ABCA7* SNPs were associated with AD in the single SNP models at P_FDR_ < 0.05 (Table 2, Tables S5 and S6 and Figure 1). Minor alleles of two of them were negatively associated, and those of the other three were positively associated with the AD risk. The pair-wise LD magnitudes measured by |r| for these SNPs were small, ranging from 0.002 to 0.382 in the AD-affected group and from 0.001 to 0.371 in the AD-unaffected group (Table S9). 

In addition, among 1225 SNP pairs mapped to this gene locus, the CompGs of 139 pairs were significantly associated with AD at P_FDR_ < 0.05. None of these 139 SNP pairs had significant interaction terms in the meta-analysis of the results from traditional interaction models (Tables S5 and S6).

**Figure 3:**
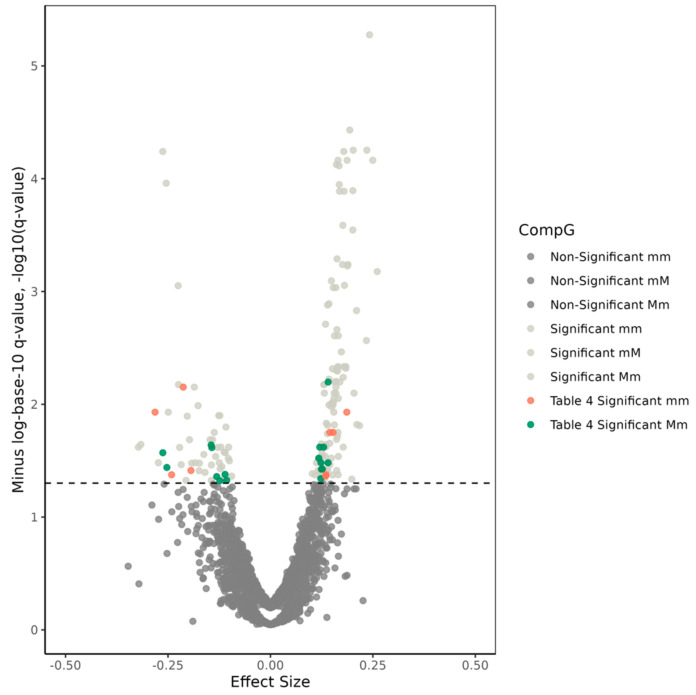
Volcano plot for compound genotype (CompG) analyses of the 1225 single-nucleotide polymorphism (SNP) pairs selected within the *ABCA7* gene region. The x-axis displays effect sizes of CompGs, while the y-axis shows minus-logarithm-base-10-transformed *q*-values, representing false-discovery rate (FDR) adjusted significance. The dashed line indicates the significance threshold, set at −log10 (*q*-value = 0.05) = 1.3. Dark-gray dots below the cutoff line represent non-significant effects for Mm, mM, and mm CompGs. Light-gray dots above the cutoff line indicate significant SNP pairs whose comprising SNPs were associated with Alzheimer’s disease (AD) individually. Red and green dots above the cutoff line denote SNP pairs whose comprising SNPs were not associated with AD individually and were presented in Table 4 and Table S5.

Twenty-four of 139 significant SNP pairs comprised of 22 SNPs that were not associated with AD in the single SNP models at P_FDR_ < 0.05 (Table 4 and Table S5 and Figure 3). Of these 24 pairs, 17 pairs had significant Mm effects, and nine pairs had significant mm effects (two pairs had significant Mm and mm effects). 

The LD magnitudes measured by |r| for these 24 SNP pairs were between 0.0007 and 0.596 in the AD-affected group and between 0.0003 and 0.630 in the AD-unaffected group. Additionally, three pairs had significantly different LD in the two groups (one with larger and two with smaller LD magnitudes in AD cases) at Bonferroni-adjusted *p* < 0.00208 (i.e., 0.05/24) (Table S10).

The remaining 115 significant SNP pairs comprised of SNPs, one or both of which were associated with AD at P_FDR_ < 0.05 in the single SNP models (Table S6 and Figure 3). We found that the SNP-AD associations can account for the CompG-AD associations for these SNP pairs as the identified CompG effects were not significantly different from the AD-associated SNP(s) main effects. Additionally, one of these 115 SNP pairs (i.e., rs4147914−rs4147937) had a significant ‘mM-Mm’ difference in the chi-square test (Table S6). This difference was partly driven by the interaction of the two SNPs as the *p*-value from the ‘mM-Mm’ comparison (*p* = 6.95 × 10^−6^) was smaller than that from the chi-square test comparing rs4147914 and rs4147937 main effects in the single SNP models (*p* = 2.80 × 10^−5^). The LD r coefficients for this SNP pair were 0.352 and 0.371 in the AD-affected and unaffected groups, respectively (Table S8).

## 4. Discussion

Our dominant allelic-effect models examining associations of individual SNPs (single SNP models) and SNP pairs (CompG models describing the effects of compound genotypes and the traditional interaction models with the SNP-by-SNP multiplicative term) in the *CLU* and *ABCA7* genes with the AD risk provided several novel insights on the genetic architecture of AD. 

First, our single SNP models showed that the AD risk was associated with six and five SNPs mapped to the *CLU* and *ABCA7* loci, respectively, with small effect sizes (β coefficients ranged from −0.156 to 0.203 and from −0.125 to 0.148, respectively) (Table 2). Of these 11 SNPs, the association between rs3752231 (*ABCA7* variant) and AD was previously reported (*p* < 6.00 × 10^−11^) [6,56,57]. In general, the magnitude of the pair-wise LD measured by the |r| between these SNPs was small to moderate. Only five of 15 *CLU* pairs and two of 10 *ABCA7* pairs had r^2^ > 0.1 (Table S9). 

Second, our analyses of the associations of 496 *CLU* and 1225 *ABCA7* SNP pairs with AD risk leveraging CompG models identified novel associations of 172 and 139 combinations of genotypes in these two loci, respectively, and AD. In contrast, the analysis of the traditional interaction models with the SNP-by-SNP term did not reveal significant interactions in the associations with AD (Tables S3–S6). Accordingly, the traditional interaction models may miss important interaction effects in the analyses of complex traits. Moreover, the CompG model describing the effects of compound genotypes on the AD risk allows transparent interpretation of the impacts of carrying minor alleles in each SNP individually (i.e., mM and Mm levels) and together (i.e., mm level). 

Most of the identified CompG-AD associations (i.e., 147 and 115 SNP pairs corresponding to the *CLU* and *ABCA7* genes, respectively) were from SNP pairs in which one or both SNPs were associated with AD individually in the single SNP models (Tables S4 and S6). Hence, the CompG-AD associations in most SNP pairs could be mainly accounted for by SNP-AD associations. However, we noticed that for four of these 262 pairs, the differences in the effects of compound heterozygotes—i.e., ‘mM-Mm’ difference—were more significant (i.e., smaller *p*-values) than the differences of the main effects of the comprising SNPs, implying partial interactions between SNPs in each pair. Thus, comparative analysis of the results from the CompG models and the single SNP models has the power to identify interaction effects characterizing differences in the effects of compound heterozygotes. 

Additionally, our CompG models showed that 25 SNP pairs mapped to the *CLU* locus (β coefficients ranged from −0.288 to 0.386) and 24 SNP pairs mapped to the *ABCA7* locus (β coefficients ranged from −0.282 to 0.186) were associated with AD, while their comprising SNPs were not associated with AD in the single SNP models (Table 3, Table 4, Tables S3 and S5).

The vast majority, 20 of 25, of the identified *CLU* CompGs were positively (i.e., adversely) associated with AD. Seven of 25 pairs composed of SNPs that had opposite directions of the effects in the single SNP models. Six of them had significant mM levels (three with protective and three with adverse effects), and one had significant adverse mm level. The other 18 pairs were from SNPs with the same directions of the effects in the single SNP models, of which six had significant mM (two with protective and four with adverse effects), eight had significant adverse mm, and four had both significant mM and mm (all with adverse effects at both mM and mm levels) (Table 3 and Table S3). 

Almost half, 13 of 24, of the AD-associated *ABCA7* CompGs showed adverse associations with AD, and the rest (11) were beneficially associated with the AD risk. Six of these 24 pairs were from SNPs that had opposite directions of the effects in the single SNP models; all of them had significant Mm levels (five with protective and one with adverse effects). The other 18 pairs, which mostly had adverse effects on the AD risk, comprised of SNPs with the same directions of effects in the single SNP models. Of them, nine had significant Mm (two with protective and seven with adverse effects), seven had significant mm (three with protective and four with adverse effects), and two had both significant Mm and mm (one with protective and the other with adverse effects at both Mm and mm levels) (Table 4 and Table S5).

The pair-wise magnitudes of LD measured by |r| for the 25 *CLU* and 24 *ABCA7* SNP pairs were small. Only six and two of these SNP pairs, respectively, had r^2^ > 0.1, implying that most of the identified SNP pairs were from SNPs that were independent (Table S10). 

Additionally, nine SNP pairs (i.e., six pairs mapped to *CLU* and three pairs mapped to *ABCA7*) had significantly different LD between the AD-affected and unaffected groups. Of them, six pairs had larger, and three pairs had smaller LD magnitudes in the AD-affected group (Table S10). The significant LD differences between AD cases and controls in the *CLU* and *ABCA7* loci are in line with the observed changes in LD patterns in the *APOE* locus [31,32,37]. 

Our findings support previous studies implicating the roles of *CLU* and *ABCA7* gene variants in AD [10,11,12,13,14,17,18]. As with previously reported complex genetic associations in the *APOE* locus, the novel CompG-AD associations identified here highlight the importance of genetic interactions in the AD risk assessment [34,36,37]. 

## 5. Conclusions

Our analyses of the AD risk identified several novel associations, mostly with small effect sizes, in previously reported AD-associated *CLU* and *ABCA7* loci. In particular, we found 49 SNP pairs in which combinations of genotypes (i.e., compound genotypes) were associated with AD, while SNPs comprising these pairs were not associated with AD individually. We also identified four partially interacting AD-associated SNP pairs, in which there were significant differences in the effects of compound heterozygotes (i.e., a major allele of one SNP and a minor allele of the other SNP in a pair) which could not be fully attributable to the main effects of the comprising SNPs. These findings expand the knowledge about the genetic architecture of AD and provide important insights into associations of combinations of SNP genotypes with AD in the *CLU* and *ABCA7* genes.

## Figures and Tables

**Table 1:** Construction of compound genotype for a pair of SNPs.

	SNP_2_ = 0	SNP_2_ = 1 or 2
**SNP_1_ = 0**	MM	Mm
**SNP_1_ = 1 or 2**	mM	mm

Abbreviations: SNP = single-nucleotide polymorphism.

**Table 2:** AD-associated SNPs in the single SNP models.

Chromosome	Position	SNP	Allele	β	Se	*p*-Value	*q*-Value	Effects
***CLU* gene**								
8	27,402,132	rs1042064	C	−0.073	0.030	1.44 × 10^−2^	3.67 × 10^−2^	−−−−
8	27,402,777	rs7341557	A	−0.156	0.038	3.98 × 10^−5^	2.03 × 10^−4^	−−−−
8	27,415,576	rs2640724	A	−0.122	0.030	3.89 × 10^−5^	2.03 × 10^−4^	−−−−
8	27,417,422	rs1873933	A	−0.119	0.034	4.30 × 10^−4^	1.64 × 10^−3^	−−−−
8	27,422,491	rs59953408	G	0.203	0.049	3.65 × 10^−5^	2.03 × 10^−4^	++++
8	27,430,506	rs7831810	G	0.110	0.032	6.15 × 10^−4^	1.88 × 10^−3^	++++
***ABCA7* gene**								
19	552,650	rs7247601	T	0.095	0.030	1.38 × 10^−3^	2.65 × 10^−2^	++++
19	1,043,638	rs3752231	T	0.148	0.030	5.76 × 10^−7^	2.88 × 10^−5^	++++
19	1,049,269	rs4147914	A	0.104	0.033	1.59 × 10^−3^	2.65 × 10^−2^	++++
19	1,065,677	rs4147937	A	−0.125	0.044	4.21 × 10^−3^	4.21 × 10^−2^	−−+−
19	1,080,189	rs2074453	C	−0.091	0.030	2.15 × 10^−3^	2.68 × 10^−2^	−−−−

Abbreviations: AD = Alzheimer’s Disease; SNP = single-nucleotide polymorphism; Position = position of SNP based on Human Genome version 38 (hg38); Alleles = effect allele of SNP; β = SNP effect size; se = standard error; *q*-value = false-discovery rate adjusted *p*-value; effects = direction of CompG effects in the ADGC, ADSP, LOAD-FBS, and UKB cohorts, respectively (+ or − symbols denote on positive or negative effects on the AD risk); ADGC = Alzheimer’s Disease Genetics Consortium initiative; ADSP = Alzheimer’s Disease Sequencing Project; LOAD-FBS = Late-Onset Alzheimer’s Disease Family-Based Study; UKB = United Kingdom Biobank.

**Table 3:** AD-associated SNP pairs in compound genotype (CompG) models mapped to the *CLU* gene region, whose comprising SNPs were not associated with AD individually.

SNP Pairs		CompG Model														
		mM					Mm					mm				
SNP1	SNP2	β	Se	*p*-Value	*q*-Value	Effects	β	Se	*p*-Value	*q*-Value	Effects	β	Se	*p*-Value	*q*-Value	Effects
rs66924402	rs10096092	−0.148	0.042	4.00 × 10^−4^	**3.41 × 10^−3^**	+−−−	−0.111	0.397	7.81 × 10^−1^	6.54 × 10^−1^	−+−+	0.011	0.037	7.66 × 10^−1^	4.24 × 10^−1^	−−−+
rs66924402	rs7845904	−0.080	0.033	1.45 × 10^−2^	**4.69 × 10^−2^**	−−−−	−0.071	0.050	1.56 × 10^−1^	4.15 × 10^−1^	−−+−	0.008	0.078	9.19 × 10^−1^	4.55 × 10^−1^	−−++
rs66924402	rs73231005	−0.121	0.045	7.07 × 10^−3^	**2.83 × 10^−2^**	+−−−	0.013	0.038	7.23 × 10^−1^	6.40 × 10^−1^	+−++	−0.001	0.041	9.84 × 10^−1^	4.70 × 10^−1^	−−++
rs66924402	rs10503815	−0.087	0.034	9.27 × 10^−3^	**3.45 × 10^−2^**	−−−−	−0.123	0.059	3.59 × 10^−2^	2.52 × 10^−1^	−−+−	−0.016	0.057	7.83 × 10^−1^	4.27 × 10^−1^	−−++
rs34319290	rs7845904	0.001	0.050	9.92 × 10^−1^	7.77 × 10^−1^	+−+−	−0.054	0.044	2.25 × 10^−1^	4.65 × 10^−1^	−−+−	0.386	0.133	3.81 × 10^−3^	**2.17 × 10^−2^**	++++
rs55986679	rs9331916	0.099	0.037	8.26 × 10^−3^	**3.11 × 10^−2^**	++++	0.079	0.038	3.68 × 10^−2^	2.54 × 10^−1^	+−++	0.024	0.054	6.54 × 10^−1^	3.93 × 10^−1^	+++−
rs55986679	rs9331888	0.103	0.042	1.38 × 10^−2^	**4.48 × 10^−2^**	++++	0.105	0.037	4.94 × 10^−3^	7.13 × 10^−2^	+−++	0.103	0.048	3.05 × 10^−2^	7.44 × 10^−2^	++++
rs17466060	rs881146	0.090	0.035	1.05 × 10^−2^	**3.76 × 10^−2^**	++++	0.094	0.061	1.20 × 10^−1^	3.80 × 10^−1^	++−+	0.109	0.064	8.99 × 10^−2^	1.34 × 10^−1^	++++
rs17466060	rs17466684	0.104	0.042	1.48 × 10^−2^	**4.74 × 10^−2^**	++++	0.058	0.052	2.57 × 10^−1^	4.88 × 10^−1^	+−++	0.086	0.054	1.09 × 10^−1^	1.48 × 10^−1^	+++−
rs17466060	rs2279591	0.118	0.046	1.02 × 10^−2^	**3.72 × 10^−2^**	+−++	0.066	0.052	2.07 × 10^−1^	4.49 × 10^−1^	+−++	0.091	0.049	6.20 × 10^−2^	1.09 × 10^−1^	++++
rs17466060	rs9331916	0.120	0.043	5.84 × 10^−3^	**2.48 × 10^−2^**	++++	0.099	0.052	5.56 × 10^−2^	3.12 × 10^−1^	+−++	0.127	0.051	1.32 × 10^−2^	**4.37 × 10^−2^**	++++
rs17466060	rs9331888	0.149	0.047	1.67 × 10^−3^	**9.44 × 10^−3^**	++++	0.156	0.053	3.02 × 10^−3^	5.31 × 10^−2^	++++	0.179	0.049	2.40 × 10^−4^	**3.77 × 10^−3^**	++++
rs17466060	rs9314349	0.161	0.052	1.81 × 10^−3^	**9.51 × 10^−3^**	++++	0.110	0.054	4.04 × 10^−2^	2.73 × 10^−1^	++++	0.125	0.049	1.11 × 10^−2^	**3.94 × 10^−2^**	++++
rs17466060	rs12549671	0.114	0.045	1.11 × 10^−2^	**3.88 × 10^−2^**	++++	0.091	0.052	7.74 × 10^−2^	3.45 × 10^−1^	++++	0.125	0.047	7.94 × 10^−3^	**3.28 × 10^−2^**	++++
rs17466060	rs36046209	0.038	0.035	2.70 × 10^−1^	3.78 × 10^−1^	+++−	−0.063	0.088	4.74 × 10^−1^	5.73 × 10^−1^	++−−	0.116	0.042	5.64 × 10^−3^	**2.71 × 10^−2^**	++++
rs4732724	rs9331888	0.068	0.042	1.07 × 10^−1^	2.10 × 10^−1^	+−++	0.082	0.045	7.06 × 10^−2^	3.35 × 10^−1^	+−++	0.112	0.043	8.78 × 10^−3^	**3.49 × 10^−2^**	++++
rs4732724	rs36046209	0.021	0.034	5.34 × 10^−1^	5.77 × 10^−1^	++++	−0.004	0.070	9.58 × 10^−1^	7.00 × 10^−1^	+−+−	0.102	0.041	1.36 × 10^−2^	**4.45 × 10^−2^**	++++
rs881146	rs9331888	−0.086	0.067	2.01 × 10^−1^	3.11 × 10^−1^	−++−	0.031	0.032	3.32 × 10^−1^	5.20 × 10^−1^	++++	0.140	0.054	9.25 × 10^−3^	**3.53 × 10^−2^**	++−+
rs881146	rs12549671	−0.053	0.057	3.50 × 10^−1^	4.48 × 10^−1^	−++−	0.002	0.032	9.55 × 10^−1^	7.00 × 10^−1^	−+−+	0.155	0.062	1.27 × 10^−2^	**4.37 × 10^−2^**	++−+
rs17466684	rs9331888	−0.288	0.101	4.23 × 10^−3^	**1.94 × 10^−2^**	−−−−	0.065	0.039	9.74 × 10^−2^	3.77 × 10^−1^	++++	0.033	0.035	3.37 × 10^−1^	2.85 × 10^−1^	+−++
rs9331888	rs73231005	0.102	0.044	1.98 × 10^−2^	5.98 × 10^−2^	++++	0.092	0.044	3.57 × 10^−2^	2.52 × 10^−1^	+−++	0.151	0.050	2.70 × 10^−3^	**1.82 × 10^−2^**	+−++
rs9331888	rs520192	0.080	0.032	1.11 × 10^−2^	**3.88 × 10^−2^**	++++	0.086	0.072	2.32 × 10^−1^	4.74 × 10^−1^	+−++	−0.004	0.059	9.47 × 10^−1^	4.60 × 10^−1^	+−−−
rs9331888	rs36046209	0.064	0.034	5.93 × 10^−2^	1.38 × 10^−1^	++++	0.070	0.045	1.21 × 10^−1^	3.80 × 10^−1^	++++	0.159	0.056	4.19 × 10^−3^	**2.29 × 10^−2^**	++++
rs73231005	rs9314349	0.121	0.048	1.18 × 10^−2^	**4.08 × 10^−2^**	++++	0.077	0.045	8.24 × 10^−2^	3.45 × 10^−1^	++++	0.075	0.046	1.03 × 10^−1^	1.43 × 10^−1^	−+++
rs73231005	rs36046209	0.003	0.035	9.24 × 10^−1^	7.67 × 10^−1^	−−++	−0.044	0.075	5.55 × 10^−1^	5.97 × 10^−1^	++−−	0.096	0.039	1.45 × 10^−2^	**4.68 × 10^−2^**	+−++

Abbreviations: AD = Alzheimer’s Disease; SNP = single-nucleotide polymorphism; mM (Mm) = CompG composed of minor allele of SNP1 (SNP2) and major allele homozygote of SNP2 (SNP1); mm = CompG composed of minor alleles of SNP1 and SNP2; β = CompG effect size; se = standard error; *q*-value = false-discovery rate adjusted *p*-value; effects = direction of CompG effects in the ADGC, ADSP, LOAD-FBS, and UKB cohorts, respectively (+ or − symbols denote on positive or negative effects on the AD risk); ADGC = Alzheimer’s Disease Genetics Consortium initiative; ADSP = Alzheimer’s Disease Sequencing Project; LOAD-FBS = Late-Onset Alzheimer’s Disease Family-Based Study; UKB = United Kingdom Biobank. Bold font denoted significant *q*-values.

**Table 4:** AD-associated SNP pairs in compound genotype (CompG) models mapped to the *ABCA7* gene cluster, whose comprising SNPs were not associated with AD individually.

SNP Pairs		CompG Model														
		mM					Mm					mm				
SNP1	SNP2	β	Se	*p*-Value	*q*-Value	Effects	β	Se	*p*-Value	*q*-Value	Effects	β	Se	*p*-Value	*q*-Value	Effects
rs2288955	rs3787011	−0.037	0.035	2.88 × 10^−1^	6.99 × 10^−1^	−−−−	−0.253	0.085	2.92 × 10^−3^	**3.63 × 10^−2^**	−−−−	−0.039	0.057	4.96 × 10^−1^	4.49 × 10^−1^	+−−−
rs12459759	rs2240160	−0.038	0.037	3.03 × 10^−1^	7.13 × 10^−1^	+−+−	−0.131	0.046	4.30 × 10^−3^	**4.37 × 10^−2^**	−−+−	−0.010	0.043	8.24 × 10^−1^	5.42 × 10^−1^	++−−
rs12459759	rs4807499	−0.082	0.047	8.18 × 10^−2^	4.30 × 10^−1^	−+−−	−0.143	0.045	1.33 × 10^−3^	**2.42 × 10^−2^**	−+−−	−0.059	0.043	1.68 × 10^−1^	2.81 × 10^−1^	++−−
rs12459759	rs2240052	−0.109	0.046	1.82 × 10^−2^	2.13 × 10^−1^	−+−−	−0.145	0.044	1.05 × 10^−3^	**2.30 × 10^−2^**	−+−−	−0.037	0.042	3.80 × 10^−1^	4.07 × 10^−1^	++−−
rs757331	rs28659974	0.089	0.054	9.77 × 10^−2^	4.60 × 10^−1^	−+++	0.123	0.043	4.66 × 10^−3^	**4.57 × 10^−2^**	++++	0.087	0.045	5.37 × 10^−2^	1.64 × 10^−1^	++++
rs3787011	rs17684161	−0.022	0.052	6.73 × 10^−1^	8.43 × 10^−1^	+−+−	−0.021	0.037	5.69 × 10^−1^	5.15 × 10^−1^	+−+−	−0.241	0.084	4.00 × 10^−3^	**4.21 × 10^−2^**	−−−−
rs3787011	rs2306718	0.008	0.060	8.92 × 10^−1^	8.79 × 10^−1^	+−+−	−0.019	0.032	5.43 × 10^−1^	5.05 × 10^−1^	++−−	−0.194	0.066	3.36 × 10^−3^	**3.86 × 10^−2^**	−−−−
rs7255896	rs28659974	0.107	0.055	5.21 × 10^−2^	3.58 × 10^−1^	++++	0.123	0.041	2.49 × 10^−3^	**3.30 × 10^−2^**	++++	0.079	0.044	7.49 × 10^−2^	2.02 × 10^−1^	++++
rs7255896	rs10439143	0.086	0.057	1.30 × 10^−1^	5.26 × 10^−1^	+−++	0.124	0.042	3.16 × 10^−3^	**3.76 × 10^−2^**	++++	0.092	0.046	4.48 × 10^−2^	1.52 × 10^−1^	++++
rs12459472	rs10439143	0.135	0.056	1.50 × 10^−2^	1.88 × 10^−1^	+−++	0.141	0.046	2.34 × 10^−3^	**3.30 × 10^−2^**	++++	0.153	0.047	1.04 × 10^−3^	**1.78 × 10^−2^**	++−+
rs12459842	rs10439143	0.204	0.064	1.49 × 10^−3^	5.62 × 10^−2^	++++	0.141	0.038	1.86 × 10^−4^	**6.35 × 10^−3^**	++++	0.088	0.049	7.08 × 10^−2^	1.95 × 10^−1^	++−+
rs351967	rs2240160	−0.050	0.038	1.94 × 10^−1^	6.06 × 10^−1^	+−−−	−0.111	0.038	3.93 × 10^−3^	**4.18 × 10^−2^**	−−−−	0.030	0.048	5.32 × 10^−1^	4.60 × 10^−1^	+−−+
rs351967	rs10413761	0.130	0.057	2.30 × 10^−2^	2.50 × 10^−1^	+−++	0.130	0.040	1.18 × 10^−3^	**2.40 × 10^−2^**	++++	0.099	0.044	2.59 × 10^−2^	1.18 × 10^−1^	++−+
rs67692521	rs10413761	0.137	0.064	3.15 × 10^−2^	2.93 × 10^−1^	+−++	0.120	0.037	1.23 × 10^−3^	**2.40 × 10^−2^**	++++	0.073	0.046	1.14 × 10^−1^	2.34 × 10^−1^	++−+
rs351976	rs10439143	0.100	0.056	7.12 × 10^−2^	4.09 × 10^−1^	+−++	0.122	0.047	9.15 × 10^−3^	7.14 × 10^−2^	++++	0.136	0.047	3.97 × 10^−3^	**4.21 × 10^−2^**	++++
rs17684161	rs10439143	0.060	0.063	3.36 × 10^−1^	7.37 × 10^−1^	−+++	0.118	0.038	1.96 × 10^−3^	**3.01 × 10^−2^**	++++	0.023	0.048	6.33 × 10^−1^	4.93 × 10^−1^	+++−
rs2930898	rs2306718	−0.037	0.040	3.47 × 10^−1^	7.40 × 10^−1^	+−−−	−0.124	0.044	5.04 × 10^−3^	**4.76 × 10^−2^**	+−−−	−0.017	0.040	6.68 × 10^−1^	5.03 × 10^−1^	−+−−
rs2930898	rs2240615	0.077	0.039	5.21 × 10^−2^	3.58 × 10^−1^	++++	0.127	0.043	3.10 × 10^−3^	**3.74 × 10^−2^**	−+++	0.074	0.042	8.14 × 10^−2^	2.09 × 10^−1^	−+++
rs2240615	rs10439143	0.122	0.057	3.23 × 10^−2^	2.93 × 10^−1^	++++	0.113	0.043	8.31 × 10^−3^	6.69 × 10^−2^	++++	0.145	0.044	1.03 × 10^−3^	**1.78 × 10^−2^**	++++
rs2240160	rs4807499	−0.130	0.048	7.21 × 10^−3^	1.26 × 10^−1^	−−−−	−0.107	0.038	4.90 × 10^−3^	**4.72 × 10^−2^**	−−−−	−0.096	0.044	2.91 × 10^−2^	1.27 × 10^−1^	+−−−
rs10413761	rs10439143	0.135	0.060	2.48 × 10^−2^	2.55 × 10^−1^	++−+	0.142	0.059	1.71 × 10^−2^	9.93 × 10^−2^	++++	0.186	0.054	5.83 × 10^−4^	**1.17 × 10^−2^**	++−+
rs28659974	rs10439143	0.079	0.056	1.61 × 10^−1^	5.72 × 10^−1^	++++	0.093	0.055	8.77 × 10^−2^	2.35 × 10^−1^	++++	0.135	0.047	4.20 × 10^−3^	**4.32 × 10^−2^**	++++
rs10411696	rs4807499	−0.241	0.081	3.11 × 10^−3^	8.97 × 10^−2^	−−−−	−0.263	0.083	1.58 × 10^−3^	**2.69 × 10^−2^**	−−−−	−0.282	0.081	5.45 × 10^−4^	**1.17 × 10^−2^**	−−−−
rs4807499	rs2269846	−0.191	0.064	3.01 × 10^−3^	8.97 × 10^−2^	−+−−	−0.164	0.064	1.00 × 10^−2^	7.28 × 10^−2^	−−−−	−0.192	0.064	2.64 × 10^−3^	**3.33 × 10^−2^**	−−−−

Abbreviations: AD = Alzheimer’s Disease; SNP = single-nucleotide polymorphism; mM (Mm) = CompG composed of minor allele of SNP1 (SNP2) and major allele homozygote of SNP2 (SNP1); mm = CompG composed of minor alleles of SNP1 and SNP2; β = CompG effect size; se = standard error; *q*-value = false-discovery rate adjusted *p*-value; effects = direction of CompG effects in the ADGC, ADSP, LOAD-FBS, and UKB cohorts, respectively (+ or − symbols denote on positive or negative effects on the AD risk); ADGC = Alzheimer’s Disease Genetics Consortium initiative; ADSP = Alzheimer’s Disease Sequencing Project; LOAD-FBS = Late-Onset Alzheimer’s Disease Family-Based Study; UKB = United Kingdom Biobank. Bold font denoted significant *q*-values.

## Data Availability

Data used in this study can be obtained from dbGaP (https://www.ncbi.nlm.nih.gov/gap/, accessed on 24 June 2023), NIAGADS (https://www.niagads.org/adsp/content/home, accessed on 24 June 2023), and the UK Biobank (https://www.ukbiobank.ac.uk/, accessed on 24 June 2023).

## Supplementary Materials

The following supporting information can be downloaded at: https://www.mdpi.com/article/10.3390/genes14091666/s1, Supplementary Information File (containing Supporting Acknowledgment and Table S1). Table S1. Basic demographic information about study participants. Table S2. Basic information on SNPs used in the association analysis. Table S3. 25 CLU AD-associated SNP pairs whose comprising SNPs were not associated with AD individually. Table S4. 147 CLU AD-associated SNP pairs whose comprising SNPs were associated with AD individually. Table S5. 24 ABCA7 AD-associated SNP pairs whose comprising SNPs were not associated with AD individually. Table S6. 115 ABCA7 AD-associated SNP pairs whose comprising SNPs were associated with AD individually. Table S7. Color-coded linkage disequilibrium (LD) matrix for 32 SNPs selected within the CLU gene in AD-affected and unaffected groups. Table S8. Color-coded linkage disequilibrium (LD) matrix for 50 SNPs selected within the ABCA7 gene in AD-affected and unaffected groups. Table S9. Linkage disequilibrium (LD) information about six CLU and five ABCA7 AD-associated SNPs in the single SNP models. Table S10. Linkage disequilibrium (LD) information about 25 CLU and 24 ABCA7 AD-associated SNP pairs.
