# Supplementary material for: Interaction Analysis Reveals Complex Genetic Associations with Alzheimer’s Disease in the CLU and ABCA7 Gene Regions
Source: Genes (Basel). 2023 Aug 23;14(9):1666. doi: 10.3390/genes14091666 (PMC10531324; doi:10.3390/genes14091666)
Supplement: Supplementary file 1 [file genes-14-01666-s001.zip › SupplementaryMaterial-R1/Supplementary Information File.pdf]

Supplementary Materials for:

Interaction analysis reveals complex genetic associations with Alzheimer's disease in the *CLU* and *ABCA7* gene regions

Alireza Nazarian*, Brandon Cook, Marissa Morado, and Alexander M. Kulminski*

Biodemography of Aging Research Unit, Social Science Research Institute, Duke University, Durham, NC, USA

*Corresponding Authors:
Alireza Nazarian and Alexander M. Kulminski
Duke University
Social Science Research Institute
Biodemography of Aging Research Unit
Erwin Mill Building, 2024 W. Main St.
Durham, NC 27705
Emails: alireza.nazarian@duke.edu and kulminsk@duke.edu

This file includes:

1. Supporting Acknowledgment
2. Table S1

Other Supplementary Materials for this manuscript include Tables S2-S10 in Excel format and provided as separate files:

1. **Table S2**: Basic information on SNPs used in the association analysis.
2. **Table S3**: 25 *CLU* AD-associated SNP pairs whose comprising SNPs were not associated with AD individually.
3. **Table S4**: 147 *CLU* AD-associated SNP pairs whose comprising SNPs were associated with AD individually.
4. **Table S5**: 24 *ABCA7* AD-associated SNP pairs whose comprising SNPs were not associated with AD individually.
5. **Table S6**: 115 *ABCA7* AD-associated SNP pairs whose comprising SNPs were associated with AD individually.
6. **Table S7**: Color-coded linkage disequilibrium (LD) matrix for 32 SNPs selected within the *CLU* gene in AD-affected and unaffected groups.
7. **Table S8**: Color-coded linkage disequilibrium (LD) matrix for 50 SNPs selected within the *ABCA7* gene in AD-affected and unaffected groups.
8. **Table S9**: Linkage disequilibrium (LD) information about six *CLU* and five *ABCA7* AD-associated SNPs in the single SNP models.
9. **Table S10**: Linkage disequilibrium (LD) information about 25 *CLU* and 24 *ABCA7* AD-associated SNP pairs.

Supporting Acknowledgment
This research was supported by Grants from the National Institute on Aging (R01AG061853, R01AG065477, and R01AG070488). The funders had no role in study design, data collection and analysis, the decision to publish, or manuscript preparation. The content is solely the responsibility of the authors and does not necessarily represent the official views of the National Institutes of Health.

This manuscript was prepared using limited access datasets obtained from dbGaP [accession numbers: phs000372.v1.p1 (ADGC), phs000572.v8.p4 (ADSP), and phs000168.v2.p2 (LOAD-FBS)], NIAGADS [accession number: NG00067 (ADSP)], and the UK Biobank [applications numbers: 60447 and 62778 (UKB)].

ADGC
Funding support for the Alzheimer's Disease Genetics Consortium (ADGC) was provided through the NIA Division of Neuroscience (U01-AG032984).

ADSP
The Alzheimer's Disease Sequencing Project (ADSP) is comprised of two Alzheimer's Disease (AD) genetics consortia and three National Human Genome Research Institute (NHGRI) funded Large Scale Sequencing and Analysis Centers (LSAC). The two AD genetics consortia are the Alzheimer's Disease Genetics Consortium (ADGC) funded by NIA (U01 AG032984), and the Cohorts for Heart and Aging Research in Genomic Epidemiology (CHARGE) funded by NIA (R01 AG033193), the National Heart, Lung, and Blood Institute (NHLBI), other National Institute of Health (NIH) institutes and other foreign governmental and non-governmental organizations. The Discovery Phase analysis of sequence data is supported through UF1AG047133 (to Drs. Schellenberg, Farrer, Pericak-Vance, Mayeux, and Haines); U01AG049505 to Dr. Seshadri; U01AG049506 to Dr. Boerwinkle; U01AG049507 to Dr. Wijsman; and U01AG049508 to Dr. Goate and the Discovery Extension Phase analysis is supported through U01AG052411 to Dr. Goate, U01AG052410 to Dr. Pericak-Vance and U01 AG052409 to Drs. Seshadri and Fornage.

Sequencing for the Follow Up Study (FUS) is supported through U01AG057659 (to Drs. PericakVance, Mayeux, and Vardarajan) and U01AG062943 (to Drs. Pericak-Vance and Mayeux). Data generation and harmonization in the Follow-up Phase is supported by U54AG052427 (to Drs. Schellenberg and Wang). The FUS Phase analysis of sequence data is supported through U01AG058589 (to Drs. Destefano, Boerwinkle, De Jager, Fornage, Seshadri, and Wijsman), U01AG058654 (to Drs. Haines, Bush, Farrer, Martin, and Pericak-Vance), U01AG058635 (to Dr. Goate), RF1AG058066 (to Drs. Haines, Pericak-Vance, and Scott), RF1AG057519 (to Drs. Farrer and Jun), R01AG048927 (to Dr. Farrer), and RF1AG054074 (to Drs. Pericak-Vance and Beecham).

The ADGC cohorts include: Adult Changes in Thought (ACT) (UO1 AG006781, UO1 HG004610, UO1 HG006375, U01 HG008657), the Alzheimer's Disease Centers (ADC) (P30 AG019610, P30 AG013846, P50 AG008702, P50 AG025688, P50 AG047266, P30 AG010133, P50 AG005146, P50 AG005134, P50 AG016574, P50 AG005138, P30 AG008051, P30 AG013854, P30 AG008017, P30 AG010161, P50 AG047366, P30 AG010129, P50 AG016573, P50 AG016570, P50 AG005131, P50 AG023501, P30 AG035982, P30 AG028383, P30 AG010124, P50 AG005133, P50 AG005142, P30 AG012300, P50 AG005136, P50 AG033514, P50 AG005681, and P50 AG047270), the Chicago Health and Aging Project (CHAP) (R01 AG11101, RC4 AG039085, K23 AG030944), Indianapolis Ibadan (R01 AG009956, P30 AG010133), the Memory and Aging Project (MAP) (R01 AG17917), Mayo Clinic (MAYO) (R01 AG032990, U01 AG046139, R01 NS080820, RF1 AG051504, P50 AG016574), Mayo Parkinson's Disease controls (NS039764, NS071674, 5RC2HG005605), University of Miami (R01 AG027944, R01

AG028786, R01 AG019085, IIRG09133827, A2011048), the Multi-Institutional Research in Alzheimer's Genetic Epidemiology Study (MIRAGE) (R01 AG09029, R01 AG025259), the National Cell Repository for Alzheimer's Disease (NCRAD) (U24 AG21886), the National Institute on Aging Late Onset Alzheimer's Disease Family Study (NIA- LOAD) (R01 AG041797), the Religious Orders Study (ROS) (P30 AG10161, R01 AG15819), the Texas Alzheimer's Research and Care Consortium (TARCC) (funded by the Darrell K Royal Texas Alzheimer's Initiative), Vanderbilt University/Case Western Reserve University (VAN/CWRU) (R01 AG019757, R01 AG021547, R01 AG027944, R01 AG028786, P01 NS026630, and Alzheimer's Association), the Washington Heights-Inwood Columbia Aging Project (WHICAP) (RF1 AG054023), the University of Washington Families (VA Research Merit Grant, NIA: P50AG005136, R01AG041797, NINDS: R01NS069719), the Columbia University HispanicEstudio Familiar de Influencia Genetica de Alzheimer (EFIGA) (RF1 AG015473), the University of Toronto (UT) (funded by Wellcome Trust, Medical Research Council, Canadian Institutes of Health Research), and Genetic Differences (GD) (R01 AG007584). The CHARGE cohorts are supported in part by National Heart, Lung, and Blood Institute (NHLBI) infrastructure grant HL105756 (Psaty), RC2HL102419 (Boerwinkle) and the neurology working group is supported by the National Institute on Aging (NIA) R01 grant AG033193.

The CHARGE cohorts participating in the ADSP include the following: Austrian Stroke Prevention Study (ASPS), ASPS-Family study, and the Prospective Dementia Registry-Austria (ASPS/PRODEM-Aus), the Atherosclerosis Risk in Communities (ARIC) Study, the Cardiovascular Health Study (CHS), the Erasmus Rucphen Family Study (ERF), the Framingham Heart Study (FHS), and the Rotterdam Study (RS). ASPS is funded by the Austrian Science Fond (FWF) grant number P20545-P05 and P13180 and the Medical University of Graz. The ASPS-Fam is funded by the Austrian Science Fund (FWF) project I904),the EU Joint Programme - Neurodegenerative Disease Research (JPND) in frame of the BRIDGET project (Austria, Ministry of Science) and the Medical University of Graz and the Steiermärkische Krankenanstalten Gesellschaft. PRODEM-Austria is supported by the Austrian Research Promotion agency (FFG) (Project No. 827462) and by the Austrian National Bank (Anniversary Fund, project 15435. ARIC research is carried out as a collaborative study supported by NHLBI contracts (HHSN268201100005C, HHSN268201100006C, HHSN268201100007C, HHSN268201100008C, HHSN268201100009C, HHSN268201100010C, HHSN268201100011C, and HHSN268201100012C). Neurocognitive data in ARIC is collected by U01 2U01HL096812, 2U01HL096814, 2U01HL096899, 2U01HL096902, 2U01HL096917 from the NIH (NHLBI, NINDS, NIA and NIDCD), and with previous brain MRI examinations funded by R01-HL70825 from the NHLBI. CHS research was supported by contracts HHSN268201200036C, HHSN268200800007C, N01HC55222, N01HC85079, N01HC85080, N01HC85081, N01HC85082, N01HC85083, N01HC85086, and grants U01HL080295 and U01HL130114 from the NHLBI with additional contribution from the National Institute of Neurological Disorders and Stroke (NINDS). Additional support was provided by R01AG023629, R01AG15928, and R01AG20098 from the NIA. FHS research is supported by NHLBI contracts N01-HC-25195 and HHSN268201500001I. This study was also supported by additional grants from the NIA (R01s AG054076, AG049607 and AG033040 and NINDS (R01 NS017950). The ERF study as a part of EUROSPAN (European Special Populations Research Network) was supported by European Commission FP6 STRP grant number 018947 (LSHG-CT-2006-01947) and also received funding from the European Community's Seventh Framework Programme (FP7/2007-2013)/grant agreement HEALTH-F4- 2007-201413 by the European Commission under the programme "Quality of Life and Management of the Living Resources" of 5th Framework Programme (no. QLG2-CT-2002- 01254). High-throughput analysis of the ERF data was supported by a joint grant from the Netherlands Organization for Scientific Research and the Russian Foundation for Basic Research (NWO-RFBR 047.017.043). The Rotterdam Study is funded by Erasmus Medical Center and

Erasmus University, Rotterdam, the Netherlands Organization for Health Research and Development (ZonMw), the Research Institute for Diseases in the Elderly (RIDE), the Ministry of Education, Culture and Science, the Ministry for Health, Welfare and Sports, the European Commission (DG XII), and the municipality of Rotterdam. Genetic data sets are also supported by the Netherlands Organization of Scientific Research NWO Investments (175.010.2005.011, 911-03-012), the Genetic Laboratory of the Department of Internal Medicine, Erasmus MC, the Research Institute for Diseases in the Elderly (014-93-015; RIDE2), and the Netherlands Genomics Initiative (NGI)/Netherlands Organization for Scientific Research (NWO) Netherlands Consortium for Healthy Aging (NCHA), project 050-060-810. All studies are grateful to their participants, faculty and staff. The content of these manuscripts is solely the responsibility of the authors and does not necessarily represent the official views of the National Institutes of Health or the U.S. Department of Health and Human Services.

The FUS cohorts include: the Alzheimer’s Disease Centers (ADC) (P30 AG019610, P30 AG013846, P50 AG008702, P50 AG025688, P50 AG047266, P30 AG010133, P50 AG005146, P50 AG005134, P50 AG016574, P50 AG005138, P30 AG008051, P30 AG013854, P30 AG008017, P30 AG010161, P50 AG047366, P30 AG010129, P50 AG016573, P50 AG016570, P50 AG005131, P50 AG023501, P30 AG035982, P30 AG028383, P30 AG010124, P50 AG005133, P50 AG005142, P30 AG012300, P50 AG005136, P50 AG033514, P50 AG005681, and P50 AG047270), Alzheimer's Disease Neuroimaging Initiative (ADNI) (U19AG024904), Amish Protective Variant Study (RF1AG058066), Cache County Study (R01AG11380, R01AG031272, R01AG21136, RF1AG054052), Case Western Reserve University Brain Bank (CWRUBB) (P50AG008012), Case Western Reserve University Rapid Decline (CWRURD) (RF1AG058267, NU38CK000480), CubanAmerican Alzheimer's Disease Initiative (CuAADI) (3U01AG052410), Estudio Familiar de Influencia Genetica en Alzheimer (EFIGA) (5R37AG015473, RF1AG015473, R56AG051876), Genetic and Environmental Risk Factors for Alzheimer Disease Among African Americans Study (GenerAAtions) (2R01AG09029, R01AG025259, 2R01AG048927), Gwangju Alzheimer and Related Dementias Study (GARD) (U01AG062602), Hussman Institute for Human Genomics Brain Bank (HIHGBB) (R01AG027944, Alzheimer's Association "Identification of Rare Variants in Alzheimer Disease"), Ibadan Study of Aging (IBADAN) (5R01AG009956), Mexican Health and Aging Study (MHAS) (R01AG018016), Multi-Institutional Research in Alzheimer's Genetic Epidemiology (MIRAGE) (2R01AG09029, R01AG025259, 2R01AG048927), Northern Manhattan Study (NOMAS) (R01NS29993), Peru Alzheimer's Disease Initiative (PeADI) (RF1AG054074), Puerto Rican 1066 (PR1066) (Wellcome Trust (GR066133/GR080002), European Research Council (340755)), Puerto Rican Alzheimer Disease Initiative (PRADI) (RF1AG054074), Reasons for Geographic and Racial Differences in Stroke (REGARDS) (U01NS041588), Research in African American Alzheimer Disease Initiative (REAAADI) (U01AG052410), Rush Alzheimer's Disease Center (ROSMAP) (P30AG10161, R01AG15819, R01AG17919), University of Miami Brain Endowment Bank (MBB), and University of Miami/Case Western/North Carolina A&T African American (UM/CASE/NCAT) (U01AG052410, R01AG028786).

The four LSACs are: the Human Genome Sequencing Center at the Baylor College of Medicine (U54 HG003273), the Broad Institute Genome Center (U54HG003067), The American Genome Center at the Uniformed Services University of the Health Sciences (U01AG057659), and the Washington University Genome Institute (U54HG003079).

Biological samples and associated phenotypic data used in primary data analyses were stored at Study Investigators institutions, and at the National Cell Repository for Alzheimer’s Disease (NCRAD, U24AG021886) at Indiana University funded by NIA. Associated Phenotypic Data used in primary and secondary data analyses were provided by Study Investigators, the NIA funded Alzheimer’s Disease

Centers (ADCs), and the National Alzheimer's Coordinating Center (NACC, U01AG016976) and the National Institute on Aging Genetics of Alzheimer's Disease Data Storage Site (NIAGADS, U24AG041689) at the University of Pennsylvania, funded by NIA This research was supported in part by the Intramural Research Program of the National Institutes of health, National Library of Medicine. Contributors to the Genetic Analysis Data included Study Investigators on projects that were individually funded by NIA, and other NIH institutes, and by private U.S. organizations, or foreign governmental or nongovernmental organizations.

LOAD-FBS

Funding support for the Late Onset Alzheimer's Disease Family Study (LOAD-FBS) was provided through the Division of Neuroscience, NIA. The LOAD-FBS includes a genome-wide association study funded as part of the Division of Neuroscience, NIA. Assistance with phenotype harmonization and genotype cleaning, as well as with general study coordination, was provided by Genetic Consortium for Late Onset Alzheimer's Disease. This manuscript was not prepared in collaboration with LOAD-FBS investigators and does not necessarily reflect the opinions or views of LOAD-FBS.

UK Biobank (UKB)

UK Biobank is a large-scale biomedical database and research resource containing genetic, lifestyle and health information from half a million UK participants. UK Biobank's database, which includes blood samples, heart and brain scans and genetic data of the 500,000 volunteer participants, is globally accessible to approved researchers who are undertaking health-related research that's in the public interest.

UK Biobank recruited 500,000 people aged between 40-69 years in 2006-2010 from across the UK. With their consent, they provided detailed information about their lifestyle, physical measures and had blood, urine and saliva samples collected and stored for future analysis.

UK Biobank's research resource is a major contributor in the advancement of modern medicine and treatment, enabling better understanding of the prevention, diagnosis and treatment of a wide range of serious and life-threatening illnesses – including cancer, heart diseases and stroke. Since the UK Biobank resource was opened for research use in April 2012, over 23,000 researchers from +90 countries have been approved to use it and more than 2,000 peer-reviewed papers that used the resource have now been published.

UK Biobank is generously supported by its founding funders the Wellcome Trust and UK Medical Research Council, as well as the British Heart Foundation, Cancer Research UK, Department of Health, Northwest Regional Development Agency and Scottish Government. The organisation has over 150 dedicated members of staff, based in multiple locations across the UK. Find out more here: http://www.ukbiobank.ac.uk

This research has been conducted using data from UK Biobank, a major biomedical database (project ID Numbers: **60447** and **62778).**

Tables

Table S1. Basic demographic information about study participants.

Dataset	N	Female, %	Case, %	Age_case (SD)	Age_control (SD)	Age_all (SD)	ε2ε2, %	ε2ε3, %	ε2ε4, %	ε3ε3, %	ε3ε4, %	ε4ε4, %
**ADGC**	4938	55.87	70.01	79.76 (7.72)	75.92 (9.57)	78.60 (8.5)	0.41	6.68	2.53	41.07	38.42	10.90
**ADSP**	2118	62.65	61.61	77.88 (11.66)	83.14 (5.67)	79.90 (10.13)	0.47	7.98	1.65	46.98	36.50	6.42
**LOAD-FBS**	3714	62.44	49.78	83.92 (8.39)	69.19 (11.53)	76.52 (12.49)	0.24	6.35	2.56	37.67	42.76	10.42
**UKB**	259580	53.33	1.16	74.41 (4.72)	71.53 (3.74)	71.56 (3.76)	0.63	12.40	2.46	58.66	23.51	2.34

Abbreviations: AD = Alzheimer's Disease; Case = AD-affected group; Control = AD-unaffected group; All = sample of cases and controls combined; ADGC = Alzheimer's Disease Genetics Consortium initiative; ADSP = Alzheimer's Disease Sequencing Project; LOAD-FBS = Late-Onset Alzheimer's Disease Family-Based Study; UKB = United Kingdom Biobank; N = number of subjects; Female, % = percentage of females; Case, % = percentage of subjects with Alzheimer's disease; Age (SD) = average age and its standard deviation; ε2ε2, % = percentage of subjects with ε2ε2 apolipoprotein E (*APOE*) genotype; ε2ε3, % = percentage of subjects with ε2ε3 *APOE* genotype; ε2ε4, % = percentage of subjects with ε2ε4 *APOE* genotype; ε3ε3, % = percentage of subjects with ε3ε3 *APOE* genotype; ε3ε4, % = percentage of subjects with ε3ε4 *APOE* genotype; ε4ε4, % = percentage of subjects with ε4ε4 *APOE* genotype.
